# Human visual explanations mitigate bias in AI-based assessment of surgeon skills

**DOI:** 10.1038/s41746-023-00766-2

**Published:** 2023-03-30

**Authors:** Dani Kiyasseh, Jasper Laca, Taseen F. Haque, Maxwell Otiato, Brian J. Miles, Christian Wagner, Daniel A. Donoho, Quoc-Dien Trinh, Animashree Anandkumar, Andrew J. Hung

**Affiliations:** 1 Department of Computing and Mathematical Sciences, California Institute of Technology, California, CA USA; 2 Center for Robotic Simulation and Education, Catherine & Joseph Aresty Department of Urology, University of Southern California, California, CA USA; 3 Department of Urology, Houston Methodist Hospital, Texas, TX USA; 4 Department of Urology, Pediatric Urology and Uro-Oncology, Prostate Center Northwest, St. Antonius-Hospital, Gronau, Germany; 5 Division of Neurosurgery, Center for Neuroscience, Children’s National Hospital, Washington DC, WA USA; 6 Center for Surgery & Public Health, Department of Surgery, Brigham and Women’s Hospital, Harvard Medical School, Boston, MA USA

**Keywords:** Biomedical engineering, Ethics, Machine learning

## Abstract

Artificial intelligence (AI) systems can now reliably assess surgeon skills through videos of intraoperative surgical activity. With such systems informing future high-stakes decisions such as whether to credential surgeons and grant them the privilege to operate on patients, it is critical that they treat all surgeons fairly. However, it remains an open question whether surgical AI systems exhibit bias against surgeon sub-cohorts, and, if so, whether such bias can be mitigated. Here, we examine and mitigate the bias exhibited by a family of surgical AI systems—SAIS—deployed on videos of robotic surgeries from three geographically-diverse hospitals (USA and EU). We show that SAIS exhibits an underskilling bias, erroneously downgrading surgical performance, and an overskilling bias, erroneously upgrading surgical performance, at different rates across surgeon sub-cohorts. To mitigate such bias, we leverage a strategy —TWIX—which teaches an AI system to provide a visual explanation for its skill assessment that otherwise would have been provided by human experts. We show that whereas baseline strategies inconsistently mitigate algorithmic bias, TWIX can effectively mitigate the underskilling and overskilling bias while simultaneously improving the performance of these AI systems across hospitals. We discovered that these findings carry over to the training environment where we assess medical students’ skills today. Our study is a critical prerequisite to the eventual implementation of AI-augmented global surgeon credentialing programs, ensuring that all surgeons are treated fairly.

## Introduction

The quality of a surgeon’s intraoperative activity (skill-level) can now be reliably assessed through videos of surgical procedures and artificial intelligence (AI) systems^1–3^. With these AI-based skill assessments on the cusp of informing high-stakes decisions on a global scale such as the credentialing of surgeons^4,5^, it is critical that they are unbiased—reliably reflecting the true skill-level of all surgeons equally^6,7^. *However, it remains an open question whether such surgical AI systems exhibit a bias against certain surgeon sub-cohorts*. Without an examination and mitigation of these systems’ algorithmic bias, they may unjustifiably rate surgeons differently, erroneously delaying (or hastening) the credentialing of surgeons, and thus placing patients’ lives at risk^8,9^.

**Fig. 1:**
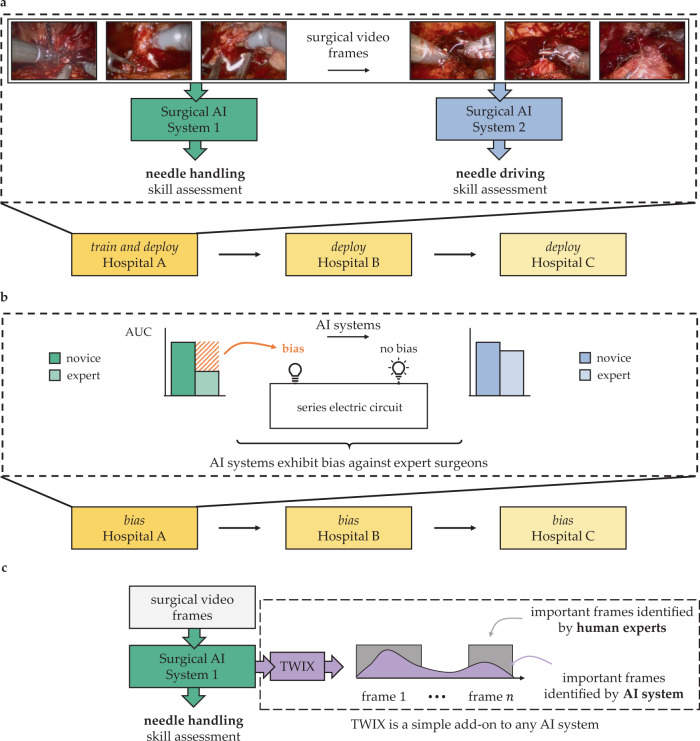
Mitigating bias of multiple surgical AI systems across multiple hospitals. **a** Multiple AI systems assess the skill-level of multiple surgical activities (e.g., needle handling and needle driving) from videos of intraoperative surgical activity. These AI systems are often deployed across multiple hospitals. **b** To examine bias, we stratify these systems' performance (e.g., AUC) across different sub-cohorts of surgeons (e.g., novices vs. experts). The bias of one of many AI systems is akin to a light bulb in an electric circuit connected in series: similar to how one defective light bulb leads to a defective circuit, one biased AI system is sufficient to disadvantage a surgeon sub-cohort. **c** To mitigate bias, we teach an AI system, through a strategy referred to as TWIX, to complement its skill assessments with predictions of the importance of video frames based on ground-truth annotations provided by human experts.

A surgeon typically masters multiple skills (e.g., needle handling and driving) necessary for surgery^10–12^. To reliably automate the assessment of such skills, multiple AI systems (one for each skill) are often developed (Fig. 1a). To test the robustness of these systems, they are typically deployed on data from multiple hospitals^13^. We argue that the bias of any one of these systems, which manifests as a discrepancy in its performance across surgeon sub-cohorts (e.g., novices vs. experts), is akin to one of many light bulbs in an electric circuit connected in series (Fig. 1b). With a single defective light bulb influencing the entire circuit, just one biased AI system is enough to disadvantage a surgeon sub-cohort. Therefore, the deployment of multiple AI systems across multiple hospitals, a common feat in healthcare, necessitates that we examine and mitigate the bias of all such systems collectively. Doing so will ethically guide the impending implementation of AI-augmented global surgeon credentialing programs^14,15^.

Previous studies have focused on algorithmic bias exclusively against *patients*, demonstrating that AI systems systematically underestimate the pain level of Black patients^16^ and falsely predict that female Hispanic patients are healthy^17^. The study of bias in video-based AI systems has also gained traction, in the context of automated video interviews^18^, algorithmic hiring^19^, and emotion recognition^20^. Previous work has not, however, investigated the bias of AI systems applied to surgical videos^21^, thereby overlooking its effect on surgeons. Further, previous attempts to mitigate such bias are either ineffective^22–24^ or are limited to a single AI system deployed in a single hospital^25–27^, casting doubt on their wider applicability. As such, previous studies do not attempt, nor demonstrate the effectiveness of a strategy, to mitigate the bias exhibited by multiple AI systems across multiple hospitals.

In this study, we examine the bias exhibited by a family of surgical AI systems—SAIS^3^—developed to assess the binary skill-level (low vs. high skill) of multiple surgical activities from videos. Through experiments on data from three geographically-diverse hospitals, we show that SAIS exhibits an *underskilling* bias, erroneously downgrading surgical performance, and an *overskilling* bias, erroneously upgrading surgical performance, at different rates across surgeon sub-cohorts. To mitigate such bias, we leverage a strategy—TWIX^28^—that teaches an AI system to complement its skill assessments with a prediction of the importance of video frames, as provided by human experts (Fig. 1c). We show that TWIX can mitigate the underskilling and overskilling bias across hospitals and simultaneously improve the performance of AI systems for all surgeons. Our findings inform the ethical implementation of impending AI-augmented global surgeon credentialing programs.

## Results

### SAIS exhibits underskilling bias across hospitals

**Fig. 2:**
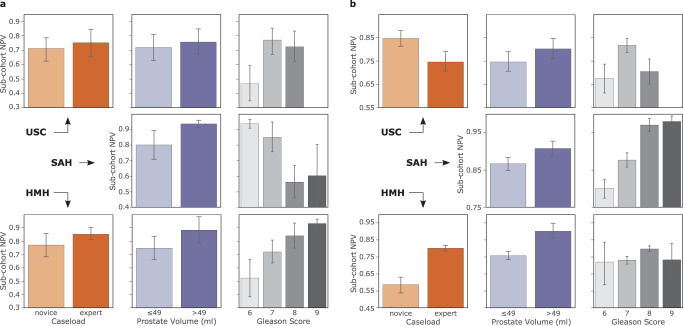
SAIS exhibits an underskilling bias across hospitals. SAIS is tasked with assessing the skill-level of **a** needle handling and **b** needle driving. A discrepancy in the negative predictive value across surgeon sub-cohorts reflects an underskilling bias. Note that SAIS is always trained on data from USC and deployed on data from St. Antonius Hospital and Houston Methodist Hospital. To examine bias, we stratify SAIS' performance based on the total number of robotic surgeries performed by a surgeon during their lifetime (caseload), the volume of the prostate gland, and the severity of the prostate cancer (Gleason score). The results are an average, and error bars reflect the standard error, across ten Monte Carlo cross-validation folds.

With skill assessment, we refer to the erroneous downgrading of surgical performance as underskilling. An underskilling bias is exhibited when such underskilling occurs at different rates across surgeon sub-cohorts. For binary skill assessment (low vs. high skill), which is the focus of our study, this bias is reflected by a discrepancy in the negative predictive value (NPV) of SAIS (see Methods, Fig. 6). We, therefore, present SAIS’ NPV for surgeons who have performed a different number of robotic surgeries during their lifetime (expert caseload >100), those operating on prostate glands of different volumes and of different cancer severity (Gleason score) (Fig. 2). Note that members of these groups are fluid as surgeons often have little say over, for example, the characteristics of the prostate gland they operate on. Please refer to the Methods section for our motivation behind selecting these groups and sub-cohorts.

We found that SAIS exhibits an underskilling bias across hospitals (see Methods for description of data, Table 2 for the number of video samples). This is evident by, for example, the discrepancy in the negative predictive value across the two surgeon sub-cohorts operating on prostate glands of different volumes (≤49 ml and >49 ml). For example, when assessing the skill-level of needle handling at USC (Fig. 2a), SAIS achieved NPV ≈ 0.71 and 0.75 for the two sub-cohorts, respectively. Such an underskilling bias consistently appears across hospitals where NPV ≈ 0.80 and 0.93 at St. Antonius Hospital (SAH), and NPV ≈ 0.73 and 0.88 at Houston Methodist Hospital (HMH). These findings extend to when SAIS assessed the skill-level of the second surgical activity of needle driving (see Fig. 2b).

#### Overskilling bias

While our emphasis has been on the underskilling bias, we demonstrate that SAIS also exhibits an overskilling bias, where it erroneously upgrades surgical performance (see Supplementary Note 2).

#### Multi-class skill assessment

Although the emphasis of this study is on binary skill assessment, a decision driven primarily by the need to inspect the fairness of a previously-developed and soon-to-be-deployed AI system (SAIS), there has been a growing number of studies focused on *multi-class* skill assessment^15^. As such, we conducted a confined experiment to examine whether such a setup, in which needle handling is identified as either low, intermediate, or high skill also results in algorithmic bias (see Supplementary Note 3). We found that both the underskilling and overskilling bias continue to extend to this setting.

### Underskilling bias persists even after controlling for potential confounding factors

Confounding factors may be responsible for the apparent underskilling bias^29,30^. It is possible that the underskilling bias against surgeons with different caseloads (Fig. 2b) is driven by SAIS’ dependence on caseload, as a proxy, for skill assessment. For example, SAIS may have latched onto the effortlessness of expert surgeons’ intraoperative activity, as opposed to the strict skill assessment criteria (see Methods), as predictive of high-skill activity. However, after controlling for caseload, we found that SAIS’ outputs remain highly predictive of skill-level (odds ratio = 2.27), suggesting that surgeon caseload, or experience, plays a relatively smaller role in assessing skill^31^ (see Methods). To further check if SAIS was latching onto caseload-specific features in surgical videos, we retrained it on data with an equal number of samples from each class (low vs. high skill) and surgeon caseload group (novice vs. expert) and found that the underskilling bias still persists. This suggests that SAIS is unlikely to be dependent on unreliable caseload-specific features.

### Examining bias across multiple AI systems and hospitals prevents misleading bias findings

With multiple AI systems deployed on the same group of surgeons across hospitals, we claim that examining the bias of only one of these AI systems can lead to misleading bias findings. Here, we provide evidence in support of this claim by focusing on the surgeon caseload group (also applies to other groups).

#### Multiple AI systems

We found that, had we examined bias for only needle handling, we would have erroneously assumed that SAIS disadvantaged novice surgeons exclusively. While SAIS did exhibit an underskilling bias against *novice* surgeons at USC when assessing the skill-level of needle handling, it exhibited this bias against *expert* surgeons when assessing the skill-level of the second surgical activity of needle driving. For example, SAIS achieved NPV ≈ 0.71 and 0.75 for novice and expert surgeons, respectively, for needle handling (Fig. 2a), whereas it achieved NPV ≈ 0.85 and 0.75 for these two sub-cohorts, for needle driving (Fig. 2b).

#### Multiple hospitals

We also found that, had we examined bias on data only from USC, we would have erroneously assumed that SAIS disadvantaged expert surgeons exclusively. While SAIS did exhibit an underskilling bias against *expert* surgeons at USC when assessing the skill-level of needle driving, it exhibited this bias against *novice* surgeons, to an even greater extent, at HMH. For example, SAIS achieved NPV ≈ 0.85 and 0.75 for novice and expert surgeons, respectively, at USC, whereas it achieved NPV ≈ 0.57 and 0.80 for these two sub-cohorts at HMH (Fig. 2b).

### TWIX mitigates underskilling bias across hospitals

Although we demonstrated, in a previous study, that SAIS was able to generalize to data from different hospitals, we are acutely aware that AI systems are not perfect. They can, for example, depend on unreliable features as a shortcut to performing a task, otherwise known as spurious correlations^32^. We similarly hypothesized that SAIS, as a video-based AI system, may be latching onto unreliable temporal features (i.e., video frames) to perform skill assessment. At the very least, SAIS could be focusing on frames which are irrelevant to the task at hand and which could hinder its performance.

**Fig. 3:**
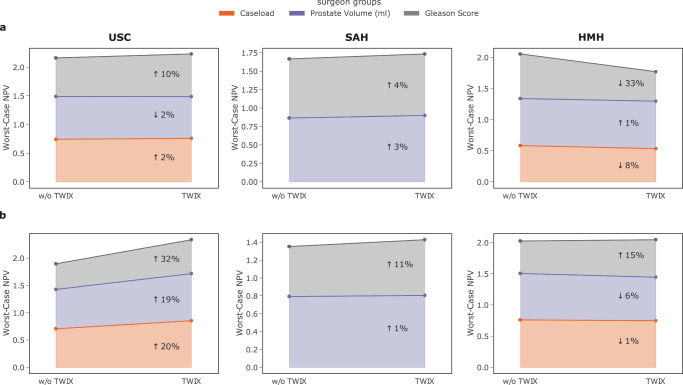
TWIX mitigates the underskilling bias across hospitals. We present the average performance of SAIS on the most disadvantaged sub-cohort (worst-case NPV) before and after adopting TWIX, indicating the percent change. An improvement (*↑*) in the worst-case NPV is considered bias mitigation. SAIS is tasked with assessing the skill-level of **a** needle handling and **b** needle driving. Note that SAIS is trained on data from USC and deployed on data from St. Antonius Hospital and Houston Methodist Hospital. Results are an average across ten Monte Carlo cross-validation folds.

To test this hypothesis, we opted for an approach that directs an AI system’s focus onto frames deemed relevant (by human experts) while performing skill assessment. The intuition is that by learning to focus on features deemed most relevant by human experts, an AI system is less likely to latch onto unreliable features in a video when assessing surgeon skill. To that end, we leverage a strategy entitled training with explanations—TWIX^28^—(see Methods). We present the performance of SAIS for the disadvantaged surgeon sub-cohorts before and after adopting TWIX when assessing the skill-level of needle handling (Fig. 3a) and needle driving (Fig. 3b).

We found that TWIX mitigates the underskilling bias exhibited by SAIS. This is evident by the improvement in SAIS’ worst-case negative predictive value for the disadvantaged surgeon sub-cohorts after having adopted TWIX. For example, when SAIS was tasked with assessing the skill-level of needle handling at USC (Fig. 3a), worst-case NPV increased by 2% for the disadvantaged surgeon sub-cohort (novice) in the surgeon caseload group (see Fig. 2 to identify disadvantaged sub-cohorts). This finding was even more pronounced when SAIS was tasked with assessing the skill-level of needle driving at USC (Fig. 3b), with improvements in the worst-case NPV by up to 32%.

**Fig. 4:**
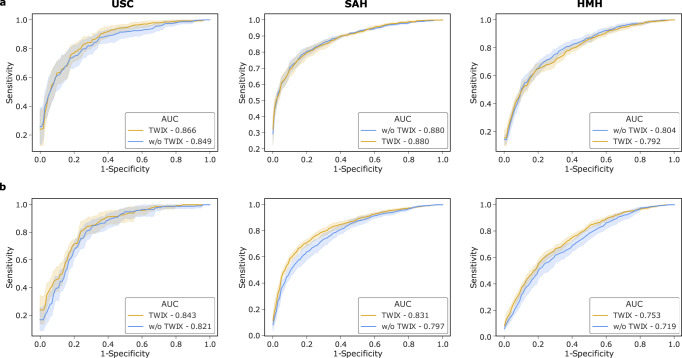
TWIX can improve AI system performance while mitigating bias across hospitals. The performance (AUC) of SAIS before and after having adopted TWIX when assessing the skill-level of **a** needle handling and **b** needle driving. Note that SAIS is trained on data from USC and deployed on data from St. Antonius Hospital and Houston Methodist Hospital. The results are an average across ten Monte Carlo cross-validation folds and the shaded area represents one standard error.

We also observed that TWIX, despite being adopted while SAIS was trained on data exclusively from USC, also mitigates bias when SAIS is deployed on data from other hospitals. This is evident by the improvements in SAIS’ performance for the disadvantaged surgeon sub-cohorts at SAH and, occasionally, at HMH. In cases where we observed a decrease in the worst-case performance, we found that this was associated with an overall decrease in the performance of SAIS (Fig. 4). We hypothesize that this reduction in performance is driven by the variability in the execution of surgical activity by surgeons across hospitals.

#### Overskilling bias

Empirically, we discovered that while various strategies mitigated the underskilling bias, they exacerbated the overskilling bias (more details in forthcoming section). In contrast, we found that TWIX avoids this negative unintended effect. Specifically, we found that TWIX also mitigates the overskilling bias (see Supplementary Note 4).

### Deploying TWIX with multiple AI systems and hospitals prevents misleading findings about its effectiveness

As with examining algorithmic bias, it is equally critical to measure the effectiveness of a bias mitigation strategy across multiple AI systems and hospitals in order to avoid misleading findings. We now provide evidence in support of this claim.

#### Multiple AI systems

We found that, had we not adopted TWIX for needle driving skill assessment, we would have underestimated its effectiveness. Specifically, while TWIX mitigated the underskilling bias at USC when SAIS assessed the skill-level of needle handling (system 1), the magnitude of this mitigation increased when SAIS assessed the skill-level of the distinct activity of needle driving (system 2). For example, for the disadvantaged surgeon sub-cohort in the caseload group, the worst-case NPV improved by 2% for needle handling (Fig. 3a) and 20% for needle driving (Fig. 3b), reflecting a 10-fold increase in the effectiveness of TWIX as a bias mitigation strategy.

#### Multiple hospitals

We found that, had we not adopted TWIX and deployed SAIS in other hospitals, we would have overestimated its effectiveness. Specifically, while TWIX mitigated the underskilling bias at USC when SAIS assessed the skill-level of needle driving, the magnitude of this mitigation decreased when SAIS was deployed on data from SAH. For example, for the disadvantaged surgeon sub-cohort in the prostate volume group, the worst-case NPV improved by 19% at USC but only by 1% at SAH (Fig. 3b).

### Baseline bias mitigation strategies induce collateral damage

A strategy for mitigating a particular type of bias can *exacerbate* another, leading to collateral damage and eroding its effectiveness. To investigate this, we adapted two additional strategies that have, in the past, proven effective in mitigating bias^33,34^. These include training an AI system with additional data (TWAD) and pre-training an AI system first with surgical videos (VPT) (see Methods for in-depth description). We compare their ability to mitigate bias to that of TWIX (Table 1 and Supplementary Note 5).

**Table 1:** Baseline strategies mitigate bias inconsistently.

	Bias mitigation strategy		
Bias	TWAD	VPT	TWIX (ours)
Underskilling	*↓* 3.7%	*↓* 7.7%	*↓* 3.0%
Overskilling	*↑* 6.7%	*↑* 7.0%	*↓* 4.0%

We report the change in the AI system’s bias (negative percent change in worst-case performance) averaged across the surgeon groups as a result of adopting distinct mitigation strategies. An improvement in the worst-case performance corresponds to a reduction in bias. Results are shown for the needle handling skill assessment system deployed on data from USC. TWAD involves training an AI system with additional data, and VPT involves pre-training the AI system with surgical videos (see Methods).

We found that while baseline strategies were effective in mitigating the underskilling bias, and even more so than TWIX, they dramatically worsened the overskilling bias exhibited by SAIS. For example, VPT almost negated its improvement in the underskilling bias (7.7%) by exacerbating the overskilling bias (7.0%). In contrast, TWIX consistently mitigated both the underskilling and overskilling bias, albeit more moderately, resulting in an average improvement in the worst-case performance by 3.0% and 4.0%, respectively. The observed consistency in TWIX’s effect on bias is an appealing property whose implications we discuss later.

### TWIX can improve AI system performance while mitigating bias across hospitals

Trustworthy AI systems must exhibit both robust and fair behavior^35^. Although it has been widely documented that mitigating algorithmic bias can come at the expense of AI system performance^36^, recent work has cast doubt on this trade-off^37–39^. We explored this trade-off in the context of TWIX, and present SAIS’ performance for all surgeons across hospitals (Fig. 4). This is reflected by the area under receiver operating characteristic curve (AUC), before and after having adopted TWIX.

We found that TWIX can improve the performance of AI systems while mitigating bias. This is evident by the improvement in the performance of SAIS both for the disadvantaged surgeon sub-cohorts (see earlier Fig. 3) and on average for all surgeons. For example, when tasked with assessing the skill-level of needle driving at USC (Fig. 3b), TWIX improved the worst-case NPV by 32%, 19%, and 20% for the surgeon groups of caseload, prostate volume, and Gleason score, respectively and thus mitigating the underskilling bias, and also improved SAIS’ performance from AUC = 0.821 → 0.843 (Fig. 4b).

### Deployment of SAIS in a training environment

**Fig. 5:**
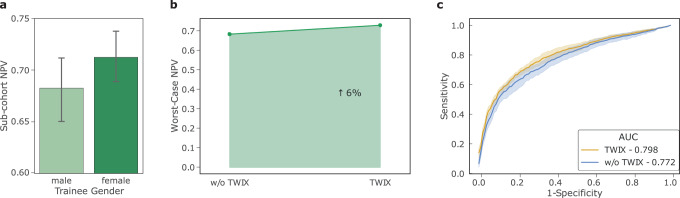
SAIS can be used today to assess the skill-level of surgical trainees. **a** SAIS exhibits an underskilling bias against male medical students when assessing the skill-level of needle handling. **b** TWIX improves the worst-case NPV, and thus mitigates the underskilling bias. **c** TWIX simultaneously improves SAIS' ability to perform skill assessment.

Our study informs the future implementation of AI-augmented surgeon credentialing programs. We can, however, begin to assess today the skills of surgical trainees in a training environment. To foster a fair learning environment for surgical trainees, it is critical that these AI-based skill assessments reflect the true skill-level of all trainees equally. To measure this, and as a proof of concept, we deployed SAIS on video samples of the needle handling activity performed by medical students without prior robotic experience on a robot otherwise used in surgical procedures (see Methods) (Fig. 5).

We discovered that our findings from when SAIS was deployed on video samples of live surgical procedures transferred to the training environment. Specifically, we first found that SAIS exhibits an underskilling bias against male medical students (Fig. 5a). Consistent with earlier findings, we also found that TWIX mitigates this underskilling bias (Fig. 5b) and simultaneously improves SAIS’ ability to assess the skill-level of needle handling (Fig. 5c).

## Discussion

Recently-developed surgical AI systems can reliably assess multiple surgeon skills across hospitals. The impending deployment of such systems for the purpose of credentialing surgeons and training medical students necessitates that they do not disadvantage any particular sub-cohort. However, until now, it has remained an open question whether such surgical AI systems exhibit algorithmic bias.

In this study, we examined and mitigated the bias exhibited by a family of surgical AI systems –SAIS –that assess the skill-level of multiple surgical activities through video. To prevent misleading bias findings, we demonstrated the importance of examining the collective bias exhibited by all AI systems deployed on the same group of surgeons and across multiple hospitals. We then leveraged a strategy—TWIX—which not only mitigates such bias for the majority of surgeon groups and hospitals, but can also improve the performance of AI systems for all surgeons.

As it pertains to the study and mitigation of algorithmic bias, previous work is limited in three main ways. First, it has not examined the algorithmic bias of AI systems applied to the data modality of surgical videos^6,40^ nor against surgeons^41,42^, thereby overlooking an important stakeholder within medicine. Second, previous work has not studied bias in the real clinical setting characterized by *multiple* AI systems deployed on the same group of surgeons and across multiple hospitals, with a single exception^43^. Third, previous work has not demonstrated the effectiveness of a bias mitigation strategy across multiple stakeholders and hospitals^33^.

When it comes to bias mitigation, we found that TWIX mitigated algorithmic bias more consistently than baseline strategies that have, in the past, proven effective in other scientific domains and with other AI systems. This consistency is reflected by a simultaneous decrease in algorithmic bias of different forms (underskilling and overskilling), of multiple AI systems (needle handling and needle driving skill assessment), and across hospitals. We do appreciate, however, that it is unlikely for a single bias mitigation strategy to be effective all the time. This reactive approach to bias mitigation might call for more of a preventative approach where AI systems are purposefully designed to exhibit minimal bias. While appealing in theory, we believe this is impractical at the present moment for several reasons. First, it is difficult to determine, during the design stage of an AI system, whether it will exhibit any algorithmic bias upon deployment on data, and if so, against whom. Mitigating bias becomes challenging when you cannot first quantify it. Second, the future environment in which an AI system will be deployed is often unknown. This ambiguity makes it difficult to design an AI system specialized to the data in that environment ahead of time. In some cases, it may even be undesirable to do so as a specialized system might be unlikely to generalize to novel data.

From a practical standpoint, we believe TWIX confers several benefits. Primarily, TWIX is a simple add-on to almost any AI system that processes temporal information and does not require any amendments to the latter’s underlying architecture. This is appealing particularly in light of the broad availability and common practice of adapting open-source AI systems. In terms of resources, TWIX only requires the availability of ground-truth importance labels (e.g., the importance of frames in a video), which we have demonstrated can be acquired with relative ease in this study. Furthermore, TWIX’s benefits can extend beyond just mitigating algorithmic bias. Most notably, when performing inference on an unseen video sample, an AI system equipped with TWIX can be viewed as explainable, as it highlights the relative importance of video frames thereby instilling trust in domain experts, and leveraged as a personalized educational tool for medical students, directing them towards surgical activity in the video that can be improved upon. These additional capabilities would be missing from other bias mitigation strategies.

We demonstrated that, to prevent misleading bias findings, it is crucial to examine and mitigate the bias of multiple AI systems across multiple hospitals. Without such an analysis, stakeholders within medicine would be left with an incomplete and potentially incorrect understanding of algorithmic bias. For example, at the national level, medical boards augmenting their decision-making with AI systems akin to those introduced here may introduce unintended disparities in how surgeons are credentialed. At the local hospital level, medical students subjected to AI-augmented surgical training, a likely first application of such AI systems, may receive unreliable learning signals. This would hinder their professional development and perpetuate existing biases in the education of medical students^44–47^. Furthermore, the alleviation of bias across multiple hospitals implies that surgeons looking to deploy an AI system in their own operating room are now less reticent to do so. As such, we recommend that algorithmic bias, akin to AI system performance, is also examined across multiple hospitals and multiple AI systems deployed on the same group of stakeholders. Doing so increases the transparency of AI systems, leading to more informed decision-making at various levels of operation within healthcare and contributing to the ethical deployment of surgical AI systems.

There are important challenges that our work does not yet address. A topic that is seldom discussed, and which we do not claim to have an answer for, is that of identifying an acceptable level of algorithmic bias. Akin to the ambiguity of selecting a performance threshold that AI systems should surpass before being deployed, it is equally unclear whether a discrepancy in performance across groups (i.e., bias) of 10 percentage points is significantly worse than that of 5 percentage points. As with model performance, this is likely to be context-specific and dependent on how costly a particular type of bias is. In our work, we have suggested that any performance discrepancy is indicative of algorithmic bias, an assumption that the majority of previous work also makes. In a similar vein, we have only considered algorithmic bias at a single snapshot in time, when the model is trained and deployed on a static and retrospectively-collected dataset. However, as AI systems are likely to be deployed over extended periods of time, where the distribution of data is likely to change, it is critical to continuously monitor and mitigate the bias exhibited by such systems over time. Analogous to continual learning approaches that allow models to perform well on new unseen data while maintaining strong performance on data observed in the past^48^, we believe *continual bias mitigation* is an avenue worth exploring.

Our study has been limited to examining the bias of AI systems which only assess the quality of two surgical skills (needle handling and needle driving). Although these skills form the backbone of suturing, an essential activity that almost all surgeons must master, they are but only a subset of all skills required of a surgeon. It is imperative for us to proactively assess the algorithmic bias of surgical AI systems once they become capable of reliably assessing a more exhaustive set of surgical skills. Another limitation is that we examine and mitigate algorithmic bias exclusively through a technical lens. However, we acknowledge that the presence and perpetuation of bias is dependent on a multitude of additional factors ranging from the social context in which an AI system is deployed, the decisions that it will inform, and the incentives surrounding its use. In this study, and for illustration purposes, we assumed that an AI system would be used to either provide feedback to surgeons about their performance or to inform decisions such as surgeon credentialing. To truly determine whether algorithmic biases, as we have defined them, translate into tangible biases that negatively affect surgeons and their clinical workflow, a prospective deployment of an AI system would be required.

Although we leveraged a bias mitigation strategy (TWIX), our work does not claim to address the key open question of *how much* bias mitigation is sufficient. Indeed, the presence of a performance discrepancy across groups is not always indicative of algorithmic bias. Some have claimed that this is the case only if the discrepancy is unjustified and harmful to stakeholders^49^. Therefore, to address this open question, which is beyond the scope of our work, researchers must appreciate the entire ecosystem in which an AI system is deployed. Moving forward, and once data become available, we look to examine (a) bias against surgeon groups which we had excluded in this study due to sample size constraints (e.g., those belonging to a particular race, sex, and ethnicity) and (b) intersectional bias^50^: that which is exhibited against surgeons who belong to multiple groups at the same time (e.g., expert surgeons who are female). Doing so could help outline whether a variant of Simpson’s paradox^51^ is at play; bias, although absent at the individual group level, may be present when simultaneously considering multiple groups. We leave this to future work as the analysis would require a sufficient number of samples from each intersectional group. We must also emphasize that a single bias mitigation strategy is unlikely to be a panacea. As a result, we encourage the community to develop bias mitigation strategies that achieve the desired effect across multiple hospitals, AI systems, and surgeon groups. Exploring the interplay of these elements, although rarely attempted in the context of algorithmic bias in medicine, is critical to ensure that AI systems deployed in clinical settings have the intended positive effect on stakeholders.

The credentialing of a surgeon is often considered a rite of passage. With time, such a decision is likely to be supported by AI-based skill assessments. In preparation for this future, our study introduces safeguards to enable fair decision-making.

## Methods

### Ethics approval

All datasets (data from USC, SAH, and HMH) were collected under Institutional Review Board (IRB) approval from the University of Southern California in which written informed consent was obtained from all participants (HS-17-00113). Moreover, the datasets were de-identifed prior to model development.

### Description of surgical procedure and activities

In this study, we focused on robot-assisted radical prostatectomy (RARP), a surgical procedure in which the prostate gland is removed from a patient’s body in order to treat cancer. With a surgical procedure often composed of sequential steps that must be executed by a surgeon, we observed the intraoperative activity of surgeons during one particular step of the RARP procedure: the vesicoureteral anastomosis (VUA). In short, the VUA is a reconstructive suturing step in which the bladder and urethra, separated by the removal of the prostate, must now be connected to one another through a series of stitches. This connection creates a tight link that should allow for the normal flow of urine postoperatively. To perform a single stitch in the VUA step, a surgeon must first grab the needle with one of the robotic arms (needle handling), push that needle through the tissue (needle driving), and then withdraw that needle on the other side of the tissue in preparation for the next stitch (needle withdrawal).

### Surgical video samples and annotations

In assessing the skill-level of suturing activity, SAIS was trained and evaluated on video samples associated with ground-truth skill assessment annotations. We now outline how these video samples and annotations were generated, and defer a description of SAIS to the next section.

#### Video samples

We collected videos of entire robotic surgical procedures from three geographically-diverse hospitals in addition to videos of medical students performing suturing activities in a laboratory environment.

##### Live robotic surgical procedures

An entire video of the VUA step (on the order of 20 min) from one surgical case was split into video samples depicting either one of the two suturing activities: needle handling and needle driving. With each VUA step consisting of around 24 stitches, this resulted in approximately 24 video samples depicting needle handling and another 24 samples depicting needle driving. To obtain these video samples, a trained medical fellow identified the start and end time of the respective suturing sub-phases. Each video sample can span 5−30 s in duration. Please refer to Table 2 for a summary of the number of video samples.

**Table 2:** Total number of videos and video samples associated with each of the hospitals and tasks.

Task	Activity	Details	Hospital	Videos	Video samples	Surgeons	Generalizing to
Skill assessment	Suturing	Needle handling	**USC**	78	912	19	Videos
			SAH	60	240	18	Hospitals
			HMH	20	184	5	Hospitals
			LAB	69	328	38	Modality
		Needle driving	**USC**	78	530	19	Videos
			SAH	60	280	18	Hospitals
			HMH	20	220	5	Hospitals

Note that we train our model, SAIS, on data exclusively shown in **bold** following a ten fold Monte Carlo cross-validation setup. For an exact breakdown of the number of video samples in each fold and training, validation, and test split, please refer to Supplementary Tables 1–6. The data from the remaining hospitals are exclusively used for inference. SAIS is always trained and evaluated on a class-balanced set of data whereby each category (e.g., low skill and high skill) contains the same number of samples. This prevents SAIS from being negatively affected by a sampling bias during training, and allows for a more intuitive appreciation of the evaluation results.

##### Training environment

To mimic the VUA step in a laboratory environment, we presented medical students with a realistic gel-like model of the bladder and urethra, and asked them to perform a total of 16 stitches while using a robot otherwise used in live surgical procedures. To obtain video samples, we followed the same strategy described above. As such, each participant’s video resulted in 16 video samples for each of the activities of needle handling, needle driving, etc. For this dataset, we only focused on needle handling (see Table 2 for a number of video samples). Note that since these video samples depict suturing activities, we adopted the same annotation strategy (described next) for these video samples and those of live surgical procedures.

#### Skill assessment annotations

A team of trained human raters (TFH, MO, and others) were tasked with viewing each video sample and annotating it with either a binary low-skill or high-skill assessment. It is worthwhile to note that, to minimize potential bias in the annotations, these raters were not privy to the clinical meta-information (e.g., surgeon caseload) associated with their surgical videos. The raters followed the strict guidelines outlined in our team’s previously-developed skill assessment tool^52^, which we outline in brief below. To ensure the quality of the annotations, the raters first went through a training process in which they annotated the same set of video samples. Once their agreement level exceeded 80%, they were allowed to begin annotating the video samples for this study. In the event of disagreements in the annotations, we followed the same strategy adopted in the original study^3^ where the lowest of all scores is considered as the final annotation.

##### Needle handling skill assessment

The skill-level of needle handling is assessed by observing the number of times a surgeon had to reposition their grasp of the needle. Fewer repositions imply a high skill-level, as they are indicative of improved surgeon dexterity and intent.

##### Needle driving skill assessment

The skill-level of needle driving is assessed by observing the smoothness with which a surgeon pushes the needle through tissue. Smoother driving implies a high skill-level, as it is less likely to cause physical trauma to the tissue.

### SAIS is an AI system for skill assessment

SAIS was recently developed to decode the intraoperative activity of surgeons based exclusively on surgical videos^3^. Specifically, it demonstrated state-of-the-art performance in assessing the skill-level of surgical activity, such as needle handling and driving, across multiple hospitals. In light of these capabilities, we used SAIS as the core AI system whose potential bias we attempted to examine and mitigate across hospitals.

#### Components of SAIS

We outline the basic components of SAIS here and refer readers to the original study for more details^3^. In short, SAIS takes two data modalities as input: RGB frames and optical flow, which measures motion in the field of view over time, and which is derived from neighboring RGB frames. Spatial information is extracted from each of these frames through a vision transformer pre-trained in a self-supervised manner on ImageNet. To capture the temporal information across frames, SAIS learns the relationship between subsequent frames through an attention mechanism. Greater attention, or importance, is placed on frames deemed more important for the ultimate skill assessment. Repeating this process for all data modalities, SAIS arrives at modality-specific video representations. SAIS aggregates these representations to arrive at a single video representation that summarizes the content of the video sample. This video representation is then used to output a probability distribution over the two skill categories (low vs. high skill).

#### Training and evaluating SAIS

As in the original study^3^, SAIS is trained on data exclusively from USC using tenfold Monte Carlo cross validation (see Supplementary Note 1). Each fold consisted of a training, validation, and test set, ensuring that surgical videos were not shared across the sets. When evaluated on data from other hospitals, SAIS is deployed on all such video samples. This is repeated for all ten of the SAIS models. As such, we report evaluation metrics as an average and standard deviation across 10 Monte Carlo cross validation folds.

### Skill assessment evaluation metrics

**Fig. 6:**
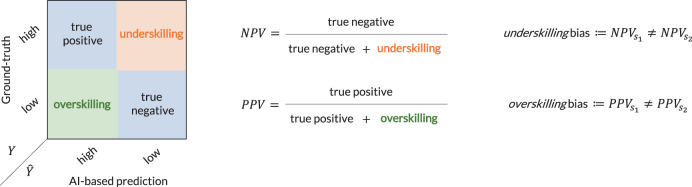
Visual definition of underskilling and overskilling bias in the context of binary skill assessment. Whereas an underskilling bias is reflected by a discrepancy in the negative predictive value of AI-based predictions across sub-cohorts of surgeons (e.g., *s*_1_ = novice and *s*_2_ = expert), and overskilling bias is reflected by a discrepancy in the positive predictive value.

When SAIS decodes surgical skills, we report the positive predictive value (PPV) defined as the proportion of AI-based high-skill assessments which are correct, and the negative predictive value (NPV) defined as the proportion of the AI-based low-skill assessments which are correct (see Fig. 6). The motivation for doing so stems from the expected use of these AI systems where their low or high-skill assessment predictions would inform decision-making (e.g., surgeon credentialing). As such, we were interested in seeing what proportion of the AI-based assessments, $\hat{Y}$, matched the ground-truth assessment, *Y*, for a given set of *S* surgeon sub-cohorts, ${\{{s}_{i}\}}_{i = 1}^{S}$

1 $${{{{\rm{PPV}}}}}_{{s}_{i}}={{{\rm{P}}}}(Y=high| {s}_{i},\hat{Y}=high)$$

2 $${{{{\rm{NPV}}}}}_{{s}_{i}}={{{\rm{P}}}}(Y=low| {s}_{i},\hat{Y}=low)$$

#### Choosing threshold for evaluation metrics

SAIS outputs the probability, *p* ∈ [0, 1], that a video sample depicts a high-skill activity. As with any probabilistic output, to make a definitive prediction (skill assessment), we had to choose a threshold, *τ*, on this probability. Whereas *p* ≤ *τ* indicates a low-skill assessment, *p* > *τ* indicates a high-skill assessment. While this threshold is often informed by previously-established clinical evidence^53^ or a desired error rate, we did not have such prior information in this setting. We also balanced the number of video samples from each skill category during the training of SAIS. As such, we chose a threshold *τ* = 0.5 for our experiments. Changing this threshold did not affect the relative model performance values across surgeon sub-cohorts, and therefore left the bias findings unchanged.

### Quantifying the different types of bias

To examine and mitigate the bias exhibited by surgical AI systems, we first require a definition of bias. Although many exist in the literature, we adopt the definition, most commonly used in recent studies^16,17,33^, as a discrepancy in the performance of an AI system for different members, or sub-cohorts, of a group (e.g., surgeons with different experience levels). The choice of performance metric ultimately depends on the type of bias we are interested in examining. In this study, we focus on two types of bias: underskilling and overskilling.

#### Underskilling

In the context of skill assessment, underskilling occurs when an AI system erroneously downgrades surgical performance, predicting a skill to be of lower quality than it actually is. Using this logic with binary skill assessment (low vs. high skill), underskilling can be quantified by the proportion of AI-based low-skill predictions ($\hat{Y}={{{\rm{low}}}}$) which should have been classified as high skill (*Y* = high). This is equivalently reflected by the negative predictive value of the AI-based predictions (see Fig. 6). While it is also possible to examine the proportion of high-skill assessments which an AI system predicts to be low-skill, amounting to the true positive rate, we opt to focus on how AI-based low-skill predictions directly inform the decision-making of an end-user.

#### Overskilling

In the context of skill assessment, overskilling occurs when an AI system erroneously upgrades surgical performance, predicting a skill to be of higher quality than it actually is. Using this logic with binary skill assessment, overskilling can be quantified by the proportion of AI-based high-skill predictions ($\hat{Y}={{{\rm{high}}}}$) which should have been classified as a low skill (*Y* = low). This is equivalently reflected by the positive predictive value of the AI-based predictions (see Fig. 6).

#### Underskilling and overskilling bias

Adopting the established definitions of bias^16,17,33^, and leveraging our descriptions of underskilling and overskilling, we define an underskilling bias as a discrepancy in the negative predictive value of AI-based predictions across sub-cohorts of surgeons, *s*_1_ and *s*_2_, for example, when dealing with two sub-cohorts (see Fig. 6). This concept naturally extends to the multi-class skill assessment setting (Supplementary Note 3). A larger discrepancy implies a larger bias. We similarly define an overskilling bias as a discrepancy in the positive predictive value of AI-based predictions across sub-cohorts of surgeons. Given our study’s focus on RARP surgical procedures, we examine bias exhibited against groups of (a) surgeons with different robotic caseloads (total number of robotic surgeries performed in their lifetime), and those operating on prostate glands of (b) different volumes, and (c) different cancer severity. We motivate this choice of groups in this next section.

### Motivation behind surgeon groups and sub-cohorts

We examined algorithmic bias against several surgeon groups. These included the volume of the prostate gland, the severity of the prostate cancer (Gleason Score), and the surgeon caseload. We chose these groups after consultation with a urologist (AH) about their relevance, and the completeness of the clinical meta-information associated with the surgical cases. It may seem counter-intuitive at first to identify surgeon groups based on, for example, the volume of the prostate gland on which they operate. After all, few surgeons make the decision to operate on patients based on such a factor. Although a single surgeon may not have a say over the volume of the prostate gland on which they operate, institution- or geography-specific patient demographics may naturally result in these groups. For example, we found that, in addition to differences in prostate volumes of patients within a hospital, there exists a difference in the distribution of such volumes across hospitals. Therefore, defining surgeon groups based on these factors still provides meaningful insight into algorithmic bias.

#### Defining surgeon sub-cohorts

In order to quantify bias as a discrepancy in model performance across sub-cohorts, we discretized continuous surgeon groups, where applicable, into two sub-cohorts. To define novice and expert surgeons, we built on previous literature which uses surgeon caseload, the total number of robotic surgeries performed by a surgeon during their lifetime, as a proxy^54–56^. As such, we define experts as having completed >100 robotic surgeries. As for prostate volume, we used the population median in the USC data to define prostate volume ≤49 ml and >49 ml. We used the population median (a) in order to have relatively balanced sub-cohorts, thus avoiding the undesirable setting where a sub-cohort has too few samples, and (b) based on previous clinical research, when available, that points to a meaningful threshold. For example, there is evidence which demonstrates that surgeons operating on large prostate glands experience longer operative times than their counterparts^57,58^. To facilitate the comparison of findings across hospitals, we used the same threshold and definition of the aforementioned sub-cohorts irrespective of the hospital.

### Implementation details to control for confounding factors

To determine the relative importance of other factors in automating skill assessment, and thus potentially acting as a source of the underskilling bias, we conducted several experiments. Without loss of generality, we conducted these experiments to check if surgeon caseload was a potential confounding factor of the observed underskilling bias. We chose this factor because we empirically observed an underskilling bias against surgeons with different caseloads and it is for a relationship to exist between caseload and the quality of surgical activity (e.g., in our dataset, 65 and 45% of assessments are considered low-skill for novice and expert surgeons, respectively).

First, after training SAIS on data from USC to assess the skill-level of needle handling, we deployed it on data from the test set (all 10 Monte Carlo cross-validation folds). Recall that SAIS returns a probabilistic value ∈ [0, 1] indicating the probability of a high-skill surgical activity. Following the setup described in Pierson et al.^59^, we train a logistic regression model where the two independent variables are SAIS’ probabilistic output and the binary surgeon caseload (0 is ≤100 cases, 1 is >100 cases) and the dependent variable is the needle handling skill-level. Across the 10 folds, we found that the coefficient of SAIS’ probabilistic output is 0.82, SD: 0.15. This suggests that caseload plays a minimal role in the assessment of surgeon skill, and is therefore an unlikely confounding factor.

To provide further evidence in support of this claim, we conducted an additional study whereby we trained SAIS after having adopted a class and sub-cohort balancing strategy. Sub-cohort balancing implies having the same number of samples from each surgeon sub-cohort in each class. Class balancing implies having the same number of samples from each class. Given a training set of data, we first performed sub-cohort balancing by inspecting each class and under-sampling data (using a random seed = 0) from the surgeon *sub-cohort* with the larger number of samples. We then performed class balancing by under-sampling data (using a random seed = 0) from the *class* with the larger number of samples. The validation and test sets in each fold remained unchanged. The intuition is that if caseload were a confounding factor, and SAIS happened to be latching onto caseload-specific features in the videos, then balancing the training data as described above should eradicate, or at least alleviate, the observed underskilling bias. However, we found the underskilling bias persisted to the same degree as before, suggesting that caseload is unlikely to be a confounding factor.

### TWIX as a bias mitigation strategy

To mitigate algorithmic bias, we adopted a strategy—TWIX^28^—which involves teaching an AI system to complement its predictions with an explanation that matches one provided by a human. Please refer to the section titled TWIX mitigates underskilling bias across hospitals for the motivation behind our adopting this approach. Next, we briefly outline the mechanics of TWIX and the process we followed for acquiring ground-truth explanation labels.

#### Mechanics of TWIX

Conceptually, TWIX is a module (i.e., a neural network), which receives a representation of data as input and returns the probability of the importance of that representation. This module is trained in a supervised manner to match the ground-truth importance label assigned to that representation. In the next section, we outline the form of these importance labels and how they are provided by human experts. As such, TWIX can be used whenever an AI system extracts representations of data and ground-truth importance labels are available.

In this study, we used TWIX alongside a video-based surgical AI system, which extracts representations of individual frames in a surgical video, where each frame was annotated as depicting either relevant or irrelevant information for the task of surgeon skill assessment. The parameters of TWIX are learned alongside those of of the main AI system (SAIS) in an end-to-end manner by optimizing an objective function with two terms; one to assess surgeon skill, and another to identify important frames. This is akin to multi-task learning. To emulate a realistic deployment setting, in which an AI system is trained on data from one hospital and deployed on data from another hospital, we learn these parameters using ground-truth explanation annotations (outlined next) exclusively from USC.

#### Acquisition of explanation labels

To train the TWIX module, as presented in this study, we require ground-truth labels reflecting the relevance of each individual frame in a surgical video. A frame is considered relevant if it depicts (or violates) the stringent set of skill assessment criteria laid out in a previously-established taxonomy^52^. Surgeon skill assessment is often based on both visual and motion cues in the surgical video. Therefore, in practice, a span of frames was identified as relevant or irrelevant, as opposed to a single frame. Specifically, a team of two trained human raters were asked to highlight the regions of time (equivalently, the span of video frames) deemed most relevant (e.g., 1 = important, 0 = not important) for assigned skill assessment.

##### Training the raters

Before embarking on the annotation process, a team of two human raters already familiar with the skill assessment taxonomy were trained to identify relevant aspects of video samples. As part of the training, each rater was then provided with the same set of training video samples to be independently annotated with explanations. Since these annotations were temporal (i.e., covering spans of frames), we quantified their agreement by calculating their intersection over union (IoU). This training process continued until the IoU >0.8, implying an 80% overlap in the frames annotated by the raters.

##### Annotating the video samples

After completing the training process, raters were asked to annotate video samples from USC. Specifically, they only annotated video samples associated with low-skill assessments because (a) it is often these assessments, which often require further intervention (e.g., for surgeon learning and improvement purposes) and (b) it is more straightforward, and less ambiguous, to annotate relevant frames for a low-skill assessment than a high-skill assessment. The former, for example, often occurs by violating one or more of the criteria identified in the skill assessment taxonomy. In the event of disagreement in the annotations of raters, we only considered the intersection of each annotation. The intuition is that we wanted to avoid annotating potentially superfluous frames as important. For example, if the first rater (R1) identified the span 0 − 5s as important whereas the second rater (R2) identified the span 1 − 6s as important, the final annotation became 1 − 5s (the intersection). We experimented with other approaches, such as considering the union, which we found to have a minimal effect on our findings.

### Measuring the effectiveness of TWIX as a bias mitigation strategy

The mitigation of algorithmic bias in surgical AI systems is critical as it reduces the rate at which groups are disadvantaged, increases the likelihood of clinical adoption by surgeons, and improves the ethical deployment of AI systems. Recall that we first defined bias as a discrepancy in the performance of an AI system across different groups (e.g., surgeon levels). Traditionally, algorithmic bias has been considered mitigated if such a discrepancy is reduced. However, this reduction, which would take place via a bias mitigation strategy, can manifest in several ways, thus obfuscating unintended effects. For example, a reduction can occur through unchanged performance for the disadvantaged group and *worsened* performance for the remaining group. It is debatable whether this behavior is desirable and ethical. An even more problematical scenario is one where the reduction in the discrepancy comes at the expense of depressed performance for all groups. This implies that each group would be further disadvantaged by the AI system.

One way to overcome these limitations is by monitoring improvements in the performance of the AI system that is achieved for the disadvantaged group, known as the worst-case performance^27^. Specifically, an improvement in the worst-case performance is a desirable objective in the context of bias mitigation as it implies that an AI system is lowering the rate with which it mistreats the disadvantaged group (e.g., through a lower error rate). We do note, though, that it remains critical to observe the effect of a bias mitigation strategy on all groups (see Discussion section). To that end, we adopted this objective when evaluating whether or not a bias mitigation strategy achieved its desired effect.

### Description of other bias mitigation strategies

In an attempt to mitigate the bias exhibited by SAIS, we also experimented with two additional strategies, which we outline below.

#### Strategy 1—training with additional data (TWAD)

Data-centric approaches, those which focus on curating a fit-for-purpose dataset with minimal annotation noise on which a model is trained, hold promise for improving the fairness of AI systems^33^. To that end, we explored the degree to which adding data to the training set would mitigate bias. Specifically, we retrieved video samples annotated (by the same team of human raters) as depicting medium skill-level and added them to the video samples from the low-skill category. The intuition was that exposing SAIS to more representative training data could help alleviate its bias. After adding these video samples to the low-skill category, we continued to balance the number of video samples from each category (low vs. high-skill). Concretely, before AD, SAIS was trained on 742 video samples, 50% of which belonged to the low-skill category. After adding data, SAIS was trained on 1522 samples with the same distribution of skill categories.

#### Strategy 2—surgical video pre-training (VPT)

Some evidence has demonstrated that exposing an AI system to vast amounts of unlabeled data before training can help mitigate bias^34^. To that end, we pre-trained a vision transformer in a self-supervised manner with images of the VUA step from all three hospitals: USC, SAH, and HMH. This amounted to ≈8 million images in total. We followed the same pre-training setup as that described in DINO^60^. In short, DINO leverages a contrastive objective function in which representations of augmented versions of an image (e.g., randomly cropped, rotated, etc.) are encouraged to be similar to one another. We conducted pre-training for two full epochs on an NVIDIA Titan RTX, lasting 48 h.

### Reporting summary

Further information on research design is available in the Nature Research Reporting Summary linked to this article.

## Supplementary information

Supplementary Information

Reporting Summary

## Data Availability

The videos of live surgical procedures from the University of Southern California, St. Antonius Hospital, and Houston Methodist Hospital are not publicly available. However, the videos and the corresponding annotations of the suturing activities performed by medical students in the training environment are available upon reasonable request from the authors.

## Code availability

All models were developed using Python and standard deeplearning libraries such as PyTorch^61^. The code for the underlying model (SAIS) can be accessed at https://github.com/danikiyasseh/SAIS and that for TWIX can be accessed at https://github.com/danikiyasseh/TWIX.
