# Supplementary material for: Human visual explanations mitigate bias in AI-based assessment of surgeon skills
Source: NPJ Digit Med. 2023 Mar 30;6:54. doi: 10.1038/s41746-023-00766-2 (PMC10063676; doi:10.1038/s41746-023-00766-2)
Supplement: Supplementary file 1 — Supplementary Information [file 41746_2023_766_MOESM1_ESM.pdf]

Supplementary Information

Human visual explanations mitigate bias in AI-based assessment of surgeon skills

Dani Kiyasseh[1,*], Jasper Laca[2], Taseen F. Haque[2], Maxwell Otiato[2], Brian J. Miles[3], Christian Wagner[4], Daniel A. Donoho[5], Quoc-Dien Trinh[6], Animashree Anandkumar[1], and Andrew J. Hung[2,*]

[1]Department of Computing and Mathematical Sciences, California Institute of Technology, CA, USA
[2]Center for Robotic Simulation and Education, Catherine & Joseph Aresty Department of Urology, University of Southern California, CA, USA
[3]Department of Urology, Houston Methodist Hospital, TX, USA
[4]Department of Urology, Pediatric Urology and Uro-Oncology, Prostate Center Northwest, St. Antonius-Hospital, Gronau, Germany
[5]Division of Neurosurgery, Center for Neuroscience, Children's National Hospital, Washington DC, USA
[6]Center for Surgery & Public Health, Department of Surgery, Brigham and Women's Hospital, Harvard Medical School, Boston, MA, USA
[*]Corresponding author: danikiy@hotmail.com, andrew.hung@med.usc.edu

Supplementary Note 1 - Datasets

Data splits

To evaluate the performance of SAIS in assessing the skill-level of surgical activity, we used 10-fold Monte Carlo cross-validation in order to evaluate the performance of SAIS. As such, in this section, we outline the training, validation, and test splits for each of those folds when SAIS was tasked with assessing the skill-level of needle handling (Table 1 left) and needle driving (Table 1 right). Please note that each sample reflects a video on the order of $10-30$ seconds in duration. With skill assessment being a binary classification task (low-skill vs. high-skill), we balance the number of samples from each class in every data split (training, validation, and test). While doing so during training ensures that the model's performance is not biased towards the majority class, balancing the classes during evaluation (e.g., on the test set) allows for a better understanding of the performance of SAIS and an appreciation of the evaluation metrics we report). For example, with a balanced test set ($50:50$ split between low-skill and high-skill activity), the area under the receiver operating characteristic curve becomes a more meaningful metric of performance.

Fold	train			validation			test		
	n	v	s	n	v	s	n	v	s
0	748	63	17	82	7	6	82	8	4
1	752	63	18	82	7	5	78	8	6
2	778	63	16	44	7	6	90	8	6
3	730	63	18	102	7	6	80	8	6
4	728	63	17	60	7	5	124	8	7
5	774	63	16	46	7	6	92	8	6
6	724	63	16	102	7	6	86	8	8
7	752	63	16	102	7	5	58	8	6
8	754	63	19	86	7	6	72	8	5
9	756	63	17	90	7	4	66	8	6

Fold	train			validation			test		
	n	v	s	n	v	s	n	v	s
0	442	63	17	42	7	6	46	8	4
1	438	63	18	42	7	5	50	8	6
2	432	63	16	44	7	6	54	8	6
3	452	63	18	42	7	6	36	8	6
4	438	62	17	38	7	5	54	8	7
5	448	63	16	30	7	6	52	8	6
6	400	63	16	62	7	6	68	8	8
7	450	63	16	54	7	5	26	8	6
8	408	63	19	48	7	6	74	8	5
9	412	63	17	58	7	4	60	8	6

Supplementary Table 1. Number of video samples (n), unique surgical videos (v), and surgeons (s) in each fold and data split at USC. We used these samples in the 10-fold Monte Carlo cross-validation setup to train and evaluate SAIS in assessing the skill-level of needle handling (left) and needle driving (right).

Number of samples in each surgeon sub-cohort

Here, we report the number of video samples used when stratifying the performance of SAIS across surgeon groups and sub-cohorts.

	caseload						prostate volume						Gleason score								
	novice			expert			≤ 49ml			> 49ml			6			7			8		
Fold	n	v	s	n	v	s	n	v	s	n	v	s	n	v	s	n	v	s	n	v	s
0	14	3	3	25	6	3	10	2	2	31	6	5	14	2	3	9	3	4	18	3	2
1	17	3	3	21	5	4	17	5	5	22	3	4	16	3	4	17	4	5	6	1	1
2	15	3	3	22	6	5	22	4	4	23	4	6	18	2	2	14	4	5	8	1	1
3	31	6	5	8	3	3	30	6	6	10	2	4	13	3	3	23	4	6	4	1	1
4	33	5	4	21	3	2	24	4	4	38	4	4	20	3	3	34	4	4	8	1	1
5	4	1	1	40	6	6	23	3	3	12	4	4	2	1	1	32	5	5	12	2	2
6	27	4	4	14	5	5	8	3	3	35	5	7	3	2	2	35	4	6	5	2	2
7	7	2	2	20	5	5	12	4	4	17	4	4	14	3	3	15	5	5			
8	16	2	2	20	7	7	22	4	5	14	4	4				21	4	5	15	4	4
9	13	3	3	17	6	4	7	2	3	26	6	6	10	1	2	15	5	6	3	1	1

Supplementary Table 2. Number of video samples (n), unique surgical videos (v), and surgeons (s) in each test fold across surgeons groups when assessing the skill-level of needle handling. We used these samples when stratifying the reliability of explanations across surgeon groups.

	caseload						prostate volume						Gleason score								
	novice			expert			≤ 49ml			> 49ml			6			7			8		
Fold	n	v	s	n	v	s	n	v	s	n	v	s	n	v	s	n	v	s	n	v	s
0	5	3	3	18	6	3	6	1	1	17	6	5	6	2	3	5	2	3	12	3	2
1	5	2	2	19	5	5	11	4	5	14	3	4	10	3	4	10	3	5	5	1	1
2	10	2	2	15	6	5	14	4	4	13	4	5	6	2	2	16	4	4	2	1	1
3	13	3	3	4	3	3	10	4	4	8	2	3	5	2	2	11	3	4	2	1	1
4	16	4	4	9	3	2	8	3	3	19	4	4	6	2	2	19	4	4	2	1	1
5	4	1	1	22	7	7	15	3	4	7	3	3				19	5	6	7	2	2
6	21	3	3	13	5	5	10	3	3	24	4	5	1	1	1	22	4	5	11	2	2
7	1	1	1	12	4	4	6	2	2	7	3	3	6	3	3	7	2	2			
8	9	2	2	28	6	6	20	4	6	17	4	3				15	4	4	22	4	4
9	7	2	2	19	7	5	9	2	4	21	6	5	5	1	2	15	5	6	7	1	1

Supplementary Table 3. Number of video samples (n), unique surgical videos (v), and surgeons (s) in each test fold across surgeons groups when assessing the skill-level of needle driving at USC. We used these samples when stratifying the reliability of explanations across surgeon groups.

	prostate volume						Gleason score											
	≤ 49ml			> 49ml			6			7			8			9		
	n	v	s	n	v	s	n	v	s	n	v	s	n	v	s	n	v	s
handling	81	18	5	39	9	4	23	7	4	66	13	5	18	5	3	13	2	2
driving	88	18	5	52	9	4	49	7	4	67	13	5	17	5	3	7	2	2

Supplementary Table 4. Number of video samples in each surgeon group from St. Antonius Hospital. We used these video samples to stratify the reliability of explanations (whether attention-based or TWIX) across surgeon sub-cohorts.

	caseload						prostate volume						Gleason score											
	novice			expert			≤ 49ml			> 49ml			6			7			8			9		
	n	v	s	n	v	s	n	v	s	n	v	s	n	v	s	n	v	s	n	v	s	n	v	s
handling	36	10	1	49	10	3	53	13	3	16	3	2	2	1	1	61	14	4	17	4	2	5	1	1
driving	57	10	1	46	10	3	71	13	3	16	3	2	4	1	1	61	14	4	31	4	2	7	1	1

Supplementary Table 5. Number of video samples in each surgeon group from Houston Methodist Hospital. We used these video samples to stratify the reliability of explanations (whether attention-based or TWIX) across surgeon sub-cohorts.

	gender					
	male			female		
	n	v	s	n	v	s
handling	64	29	17	100	40	21

Supplementary Table 6. Number of video samples in each group from the laboratory environment. We used these video samples to stratify the reliability of explanations (whether attention-based or TWIX) across sub-cohorts.

Supplementary Note 2 - Overskilling bias

SAIS exhibits an overskilling bias

We showed that SAIS exhibits an underskilling bias, erroneously downgrading surgical performance. Here, we provide evidence that SAIS also exhibits an overskilling bias, erroneously upgrading surgical performance (Supplementary Fig. 1). This is evident by the discrepancy in the PPV for the different surgeon sub-cohorts.

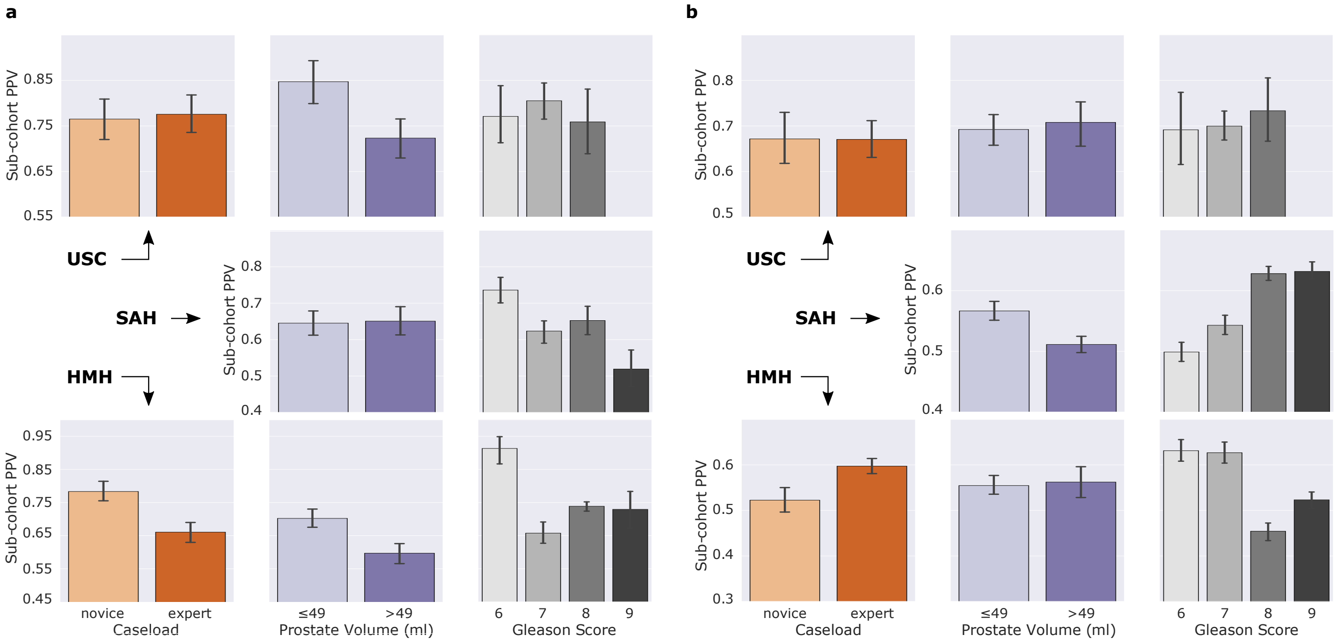

Supplementary Figure 1. SAIS exhibits an overskilling bias across hospitals. SAIS is tasked with assessing the skill-level of **a,** needle handling and **b,** needle driving. A discrepancy in the negative predictive value across surgeon sub-cohorts reflects an underskilling bias. Note that SAIS is always trained on data from USC and deployed on data from St. Antonius Hospital and Houston Methodist Hospital. To examine bias, we stratify SAIS' performance based on the total number of robotic surgeries performed by a surgeon during their lifetime (caseload), the volume of the prostate gland, and the severity of the prostate cancer (Gleason score). The results are an average across 10 folds and the error bars represent one standard error.

Supplementary Note 3 - Multi-class skill assessment

Multi-class skill assessment systems continue to exhibit bias

We demonstrated that a binary surgeon skill assessment system (SAIS) exhibits both an underskilling and overskilling bias.

Implementation details Here, we train SAIS from scratch in order to perform multi-class skill assessment (low vs. intermediate vs. high skill) and assess the degree of its algorithmic bias. This is made possible by trained raters who had previously provided such annotations in the past after following the strict set of criteria in the skill assessment taxonomy. For training and evaluation of the AI system, we follow the same exact strategy as that outlined in the Methods section. Namely, we adopt a 10-fold Monte Carlo cross validation setup where we balance the number of video samples from each class (both during training and evaluation).

Evaluation metrics We do note that because this is a multi-class setup, we have to be careful about the evaluation metrics used to quantify the underskilling and overskilling bias. To remain consistent with their definitions (see Results), we use the shown elements of the confusion matrix (Supplementary Fig. 2, left) to calculate the degree to which underskilling or overskilling occurs. In other words, we define underskilling as having occurred if the AI system predicts a skill lower than the true skill. For example, predicting a low skill for a true intermediate or high skill, and predicting an intermediate skill for a true high skill. These correspond to the *upper triangular* region of the confusion matrix. By applying the same logic to overskilling, we can see that the rate with which it occurs can be gleaned from the *lower triangular* portion of the confusion matrix. We normalize these values based on the total number of predictions for a particular surgeon sub-cohort and present these values in Supplementary Fig. 2 (right).

Findings We found that such a multi-class system continues to exhibit an underskilling and overskilling bias, emphasizing the need for bias mitigation strategies to alleviate this issue. Note that the degree of bias exhibited by this system cannot be directly compared to the bias exhibited by the binary skill assessment system for several reasons. First and foremost, they are both evaluated on distinct datasets (due to the inclusion of video samples with an intermediate skill label). Second, although the evaluation metrics are similar in spirit in that they both capture either an underskilling or overskilling bias, they remain distinct from one another (e.g., discrepancy in underskilling in the multi-class setting, and discrepancy in negative predictive value in binary setting).

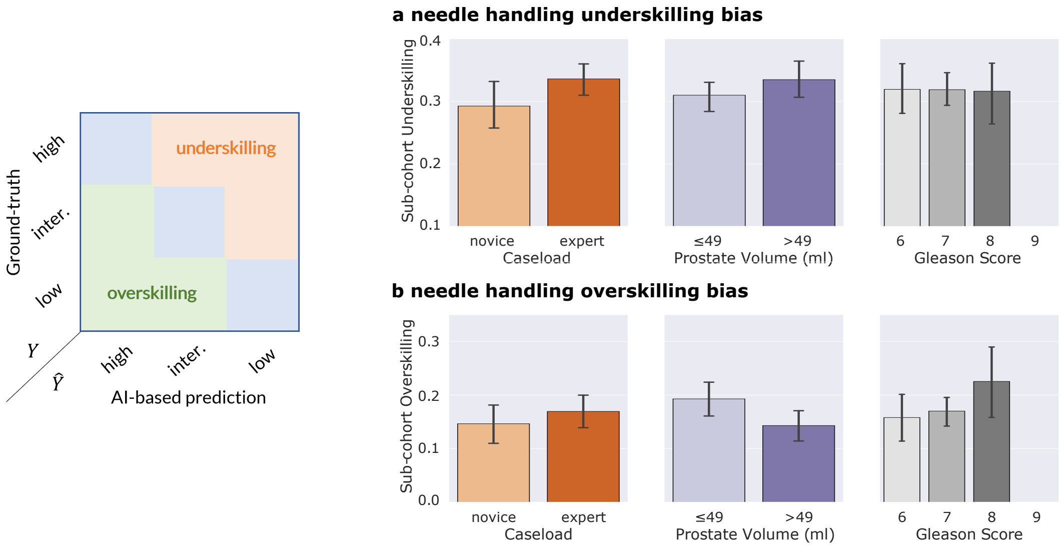

Supplementary Figure 2. Multi-class skill assessment system continues to exhibit algorithmic bias. (left) A confusion matrix reflecting underskilling and overskilling predictions for multi-class skill assessment. (right) SAIS is tasked with assessing the skill-level of needle handling on data from USC. A discrepancy in the rate of underskilling reflects an underskilling bias whereas a discrepancy in the rate of overskilling reflects an overskilling bias (see Evaluation metrics for details). To examine bias, we stratify SAIS' performance based on the total number of robotic surgeries performed by a surgeon during their lifetime (caseload), the volume of the prostate gland, and the severity of the prostate cancer (Gleason score). The results are an average across 10 folds and the error bars represent one standard error.

Supplementary Note 4 - Overskilling bias

TWIX can mitigate overskilling bias across hospitals

We demonstrated that TWIX can mitigate the underskilling bias exhibited by SAIS. Having shown that SAIS also exhibits an overskilling bias, we explored whether TWIX can also mitigate this bias. To do so, we present the percent change in the worst-case PPV after adopting TWIX during the training of SAIS (Supplementary Fig. 3). We found that TWIX can mitigate the overskilling bias across hospitals. This is evident by the improvement in the worst-case PPV for the different surgeon groups at USC and SAH.

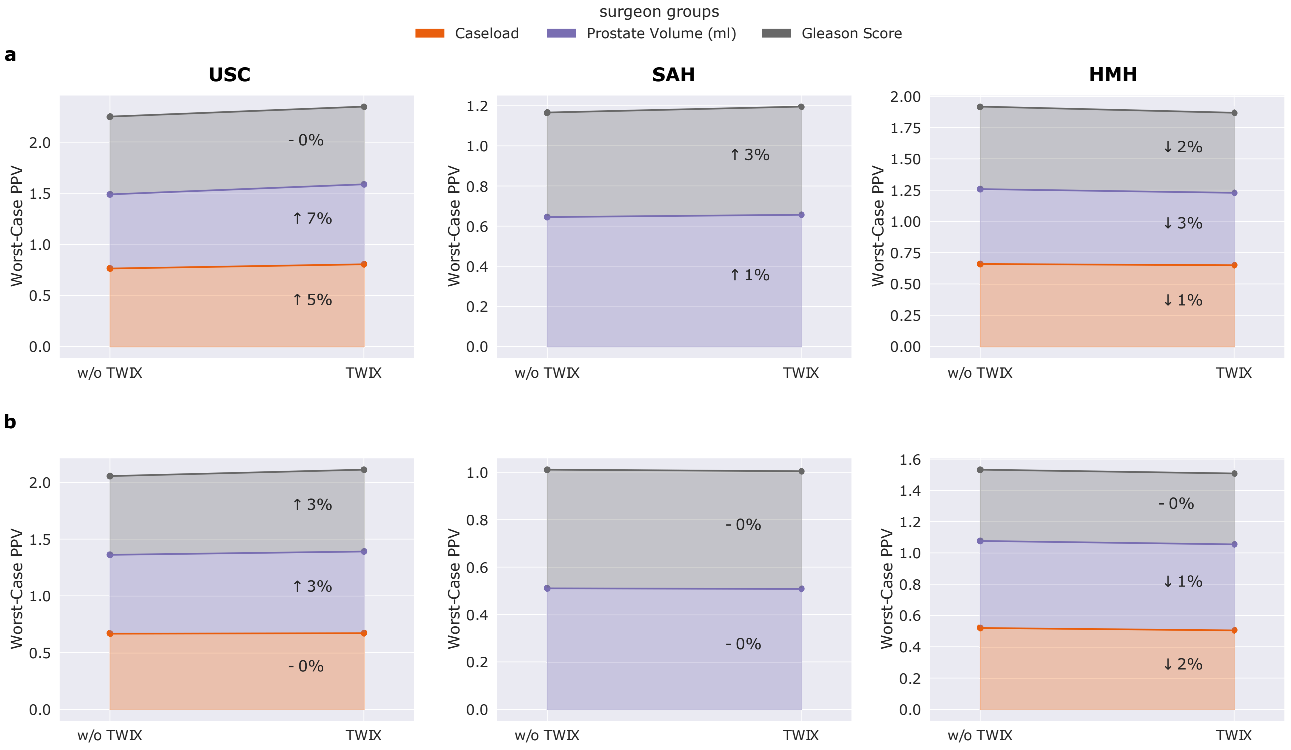

Supplementary Figure 3. TWIX mitigates the overskilling bias across hospitals. We present the average performance of SAIS on the most disadvantaged sub-cohort (worst-case NPV) before and after adopting TWIX, indicating the percent change. An improvement (↑) in the worst-case NPV is considered bias mitigation. SAIS is tasked with assessing the skill-level of **a,** needle handling and **b,** needle driving. Note that SAIS is trained on data from USC and deployed on data from St. Antonius Hospital and Houston Methodist Hospital. Results are an average across 10 folds.

Supplementary Note 5 - Effectiveness of other bias mitigation strategies

We measured the effectiveness of two additional strategies in mitigating the bias exhibited by SAIS. These two strategies, additional data (AD) and surgical video pre-training (VP), are described in detail in the Methods section. We present the change in the worst-case performance (either NPV or PPV) before and after adopting these two strategies for the task of needle handling skill assessment at USC (Supplementary Fig. 4).

We found that while AD and VP do indeed mitigate the underskilling bias, and even more so than TWIX (see Results), they exacerbate the overskilling bias. This is evident by the improvement in the worst-case NPV and a simultaneous reduction in the worst-case PPV after adopting these strategies. These findings emphasize the importance of considering the collateral damage of a bias mitigation strategy: how does it negatively affect other types of bias?

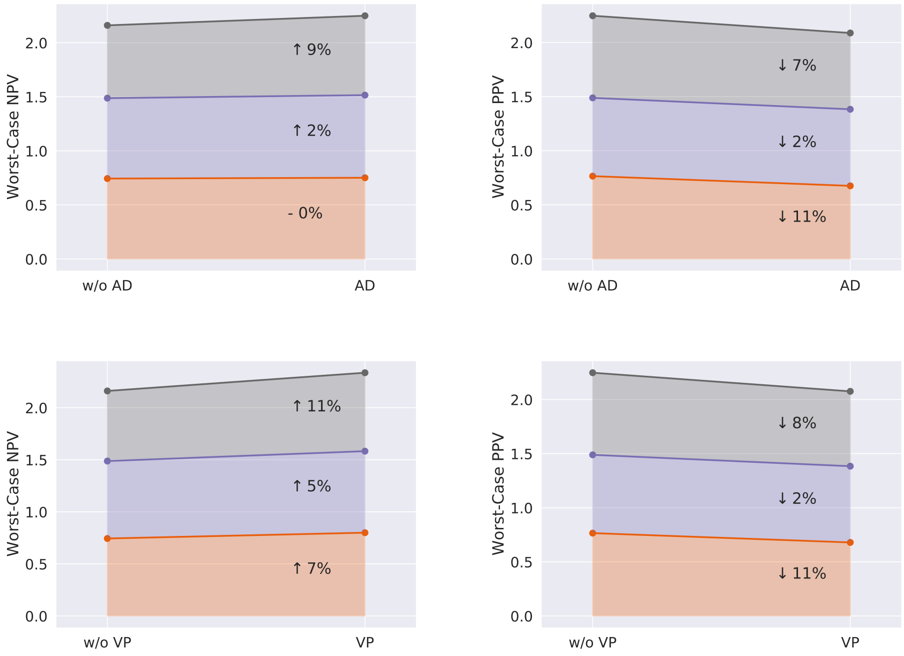

Supplementary Figure 4. Other bias mitigation strategies mitigate underskilling bias yet with collateral damage. We present the average performance of SAIS on the most disadvantaged sub-cohort (worst-case NPV and PPV) before and after adopting two different bias mitigation strategies (top: AD and bottom: VP), indicating the percent change. An improvement (↑) in the worst-case NPV or PPV is considered bias mitigation. SAIS is tasked with assessing the skill-level of needle handling at USC. Results are an average across 10 folds.
